# Prospective Evaluation of the Prediction Score for a Mild Course of Crohn’s Disease (PreMiCC) in Newly Diagnosed Patients With Crohn’s Disease: The PROGNOS Study

**DOI:** 10.1093/ibd/izae086

**Published:** 2024-04-22

**Authors:** Wolfgang Kruis, Bernd Bokemeyer, Petra Jessen, Mark Hoesl, Michael Mroß, Julia Morgenstern, Birgitta Reimers, Nike Müller-Grage, Ludger Leifeld

**Affiliations:** Internal Medicine, Protestant Hospital, Cologne, Germany; Department of Internal Medicine, Interdisciplinary Crohn Colitis Centre, Minden, Germany; Clinic of General Internal Medicine I, University Hospital Schleswig-Holstein, Campus Kiel, Kiel, Germany; Gastroenterology Practice, Kiel-Altenholz, Germany; Gastroenterology Practice Clinic, Nuremberg, Germany; Gastroenterology Practice, Berlin, Germany; Internal Medicine, Protestant Hospital, Cologne, Germany; Department of Internal Medicine, Ferring Arzneimittel GmbH, Kiel, Germany; Department of Internal Medicine, Ferring Arzneimittel GmbH, Kiel, Germany; Department of Internal Medicine, St. Bernward Hospital, Hildesheim, Germany

**Keywords:** Crohn’s disease, prediction score, validation, mild course

## Abstract

**Background and Aims:**

The course of Crohn’s disease (CD) is highly variable. The Prospektive Evaluation eines Score zur Vorhersage eines milden Verlaufsbei neu diagnostizierten Morbus Crohn-Patienten in gastroenterologischen Fachpraxen (PROGNOS) study aimed to determine the frequency of a mild disease course and validate a proposed prediction score.

**Methods:**

The PROGNOS study is a prospective study of CD patients who were newly diagnosed and, except for 1 course of 5-aminosalicylic acid or steroids for ≤10 days, therapy-naïve. Among other predefined inclusion criteria, the initial diagnosis had to be made ≤6 weeks before enrollment. All inception cohort patients were diagnosed and screened consecutively in participating gastroenterology practices in Germany specialized in inflammatory bowel disease. All screened CD patients were scored and, if possible, included in the study for up to 5 years (NCT02193048).

**Results:**

A total of 201 CD patients were included in the study (43.3% male; mean age 33 years, mean follow-up 38 months). Altogether, 29.5% of the patients had a mild course at 36 months. Among those with a score ≤2, therapy escalation at 36 months was necessary for only 24.2%, whereas in the group with a score >2, therapy escalation was necessary for 70.2% of patients. In the Kaplan-Meier curve showing time to therapy escalation in the 2 groups, there was a pronounced and statistically significant divergence of the curves starting at 3 months and extending to 48 months (*P* < .001).

**Conclusions:**

In this prospective study, about 30% of incident CD patients had a mild disease course. Our suggested PreMiCC (prediction score for a mild course of Crohn’s disease) successfully predicted this.

## Graphical Abstract

**Graphical Abstract:**
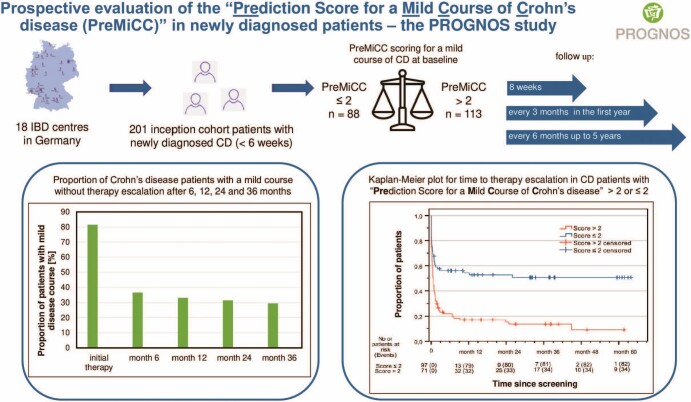

**Key Messages**

**What is already known?**

The course of Crohn’s disease (CD) is highly variable and requires a selective approach to treatment, with some patients experiencing complications and others having a milder disease course.

**What is new here?**

In our prospective PROGNOS study, 29.5% of the CD patients had a mild course at 36 months, which we were able to predict with our PreMiCC (prediction score for a mild course of Crohn’s disease), validating the score in our German cohort.

**How can this study help patient care?**

In incident CD, approximately 30% of patients were found to have a mild disease course without needing immunosuppressive/biological therapy. Predicting mild disease courses will facilitate selecting therapy for such patients.

## Introduction

The course of Crohn’s disease (CD) is highly variable and requires a selective approach to treatment, with some patients experiencing complications and others having a milder disease course. Indeed, while some patients with a severe course of disease appear to benefit from top-down therapy,^1,2^ a substantial proportion have a rather mild course and can be treated successfully with modified conventional therapy comprising only mesalazine (or the short-term use of steroids as needed) and careful monitoring.^3^

In patients at risk of complications, extended immunosuppressive/advanced therapy can prevent chronic intestinal damage.^4,5^ For this reason, it is important to consider, early in the course of disease, the potential benefits of such therapy and, where indicated, to find the appropriate window of opportunity to initiate it.^6^ Several studies have tried to define this window by identifying risk factors predictive of a severe course.^7^ At the same time, it is important to avoid overtreatment in other cases, both to reduce undesirable side effects and to avoid unnecessary costs to the healthcare system.

The proportion of CD patients with a mild course of disease and without relevant complications has been reported in the literature to range from approximately 20% to 30%.^8-12^ The European Crohn’s and Colitis Organization (ECCO) working group in 2017 used the term “nonprogressive course” to describe a mild or uncomplicated disease course.^13^ For these patients, the updated ECCO guideline from 2020 recommends only a short cycle of therapy with budesonide alongside a watch-and-wait approach.^3^ The search for prognostic criteria for this mild course, however, has not been successful in the several studies on genetic, serologic, or other biomarkers of CD that have been conducted to date.^14-20^ In the following, we prefer to use the term “mild course” instead of the ECCO proposal “nonprogressive course,” given that the latter term could trigger a false association with the general progression of the disease.

In a monocentric pilot study in Germany,^21^ we showed that among patients with newly diagnosed CD, 18% were able to achieve long-term remission with mesalazine alone. In 2012, we extended our search for such factors by conducting a multicenter, retrospective survey of patients who were both newly diagnosed with CD and treated in participating inflammatory bowel disease (IBD)–experienced gastroenterology practices.^11^ In that study, which documented the disease course of 162 patients, 29% were found to have a mild course with no need for therapy escalation. Several of the criteria significantly associated with a mild course were subsequently used to develop a score based on age at disease onset, C-reactive protein (CRP), endoscopic severity, perianal involvement, and the combined incidence of complications: the PreMiCC (prediction score for a mild course of Crohn’s disease). Yanai et al^12^ also conducted a study on the early indolent course of CD in newly diagnosed patients, and 33% had a mild course that did not require surgery or therapy escalation with immunomodulators or biologics. Based on their findings, they proposed a digital calculator that uses a multivariate Cox regression model with several variables (body mass index, white blood cell count, vitamin B12, and alanine aminotranasferase) to assess the probability of achieving such an indolent course. Most treatment decisions, however, must be made early in real-world situations, and the digital calculator has several features that make it impractical in this regard, including a lack of clear cutoff values.

Therefore, the aim of the present prospective, real-world, observational study was to test and validate our PreMiCC, which relies on simple and readily available clinical parameters to predict a mild, nonprogressive course of CD.^11^

## Methods

### Study Population and Design

The Prospektive Evaluation eines Score zur Vorhersage eines milden Verlaufsbei neu diagnostizierten Morbus Crohn-Patienten in gastroenterologischen Fachpraxen (PROGNOS) study was a prospective, multicenter, noninterventional study of an inception cohort of CD patients in Germany that was conducted between 2014 and 2021. According to German law, noninterventional means that the decision about the diagnostic procedures or therapeutic concepts to be implemented in each patient is made only by the treating physician according to clinical needs. A total of 18 gastroenterology practices in Germany took part in the study, each of which was specialized in IBD. All practices had previously confirmed that, based on the patient’s disease severity and individual circumstances, they would consider the full spectrum of therapeutic options, including aminosalicylates, immunosuppressants, biologics, and steroids.

To be included in the study, patients had to (1) receive a confirmed new diagnosis of CD according to the German and ECCO guidelines on CD^3,22^ and based on the findings of clinical, endoscopic, histologic, and laboratory parameters (in individual cases with diagnostic uncertainty after the first colonoscopy, a second colonoscopy was performed within a few weeks to confirm the diagnosis); (2) receive this diagnosis <6 weeks before their inclusion in the study; (3) be previously untreated or have received only short-term oral steroid (budesonide or prednisolone) or oral mesalazine treatment for a maximum of 10 days; and (4) provide written informed consent. Emergency cases requiring immediate intensified therapy were excluded, as were patients with the immediate need for inpatient treatment, a clear indication for surgical intervention, or complicating infectious diseases.

**Figure 1.**
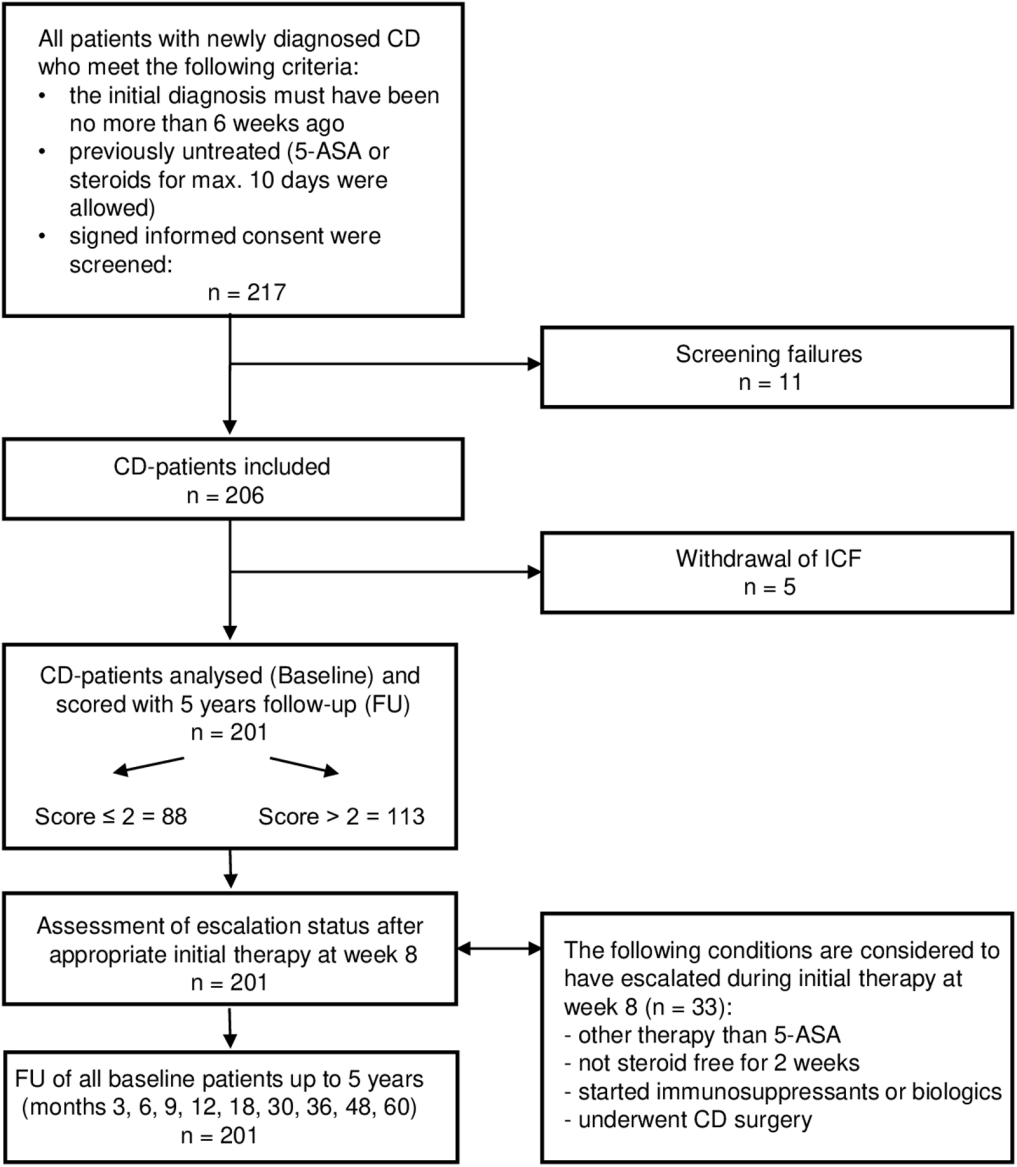
Flow diagram of the PROGNOS study. 5-ASA, 5-aminosalicylic acid; CD, Crohn’s disease; ICF, Informed Consent Form; PROGNOS, Prospektive Evaluation eines Score zur Vorhersage eines milden Verlaufsbei neu diagnostizierten Morbus Crohn-Patienten in gastroenterologischen Fachpraxen.

In the participating practices, all newly diagnosed CD patients were consecutively screened to determine whether they met the inclusion criteria described previously. In total, 217 patients fulfilled the criteria, could be classified at baseline using the PreMiCC, and were subsequently considered eligible for participation in the study. After 11 patients were excluded due to screening errors and 5 patients withdrew their consent, the final study cohort consisted of 201 patients. A study flow diagram is provided in Figure 1.

### Assessment

In this prospective study, patients were classified at baseline using the PreMiCC score.^11^ The score, which ranges from 0 to 6 points, incorporates 5 parameters: age, CRP, endoscopic findings, perianal lesions, and complications such as extraintestinal manifestations, stenosis/fistula, or fever (Table 1). Patients with a score of ≤2 points were classified as having a mild course of disease. The assessment was performed by the attending physicians with the support of the study nurses. The study team, including physicians, received training on how to calculate the score before the study commenced.

**Table 1.** Parameters of the PreMiCC score

Parameter	Results	Points
Age at first diagnosis, y	≤40	1
	>40	0
CRP, mg/dL	<2	0
	2-4	1
	>4	2
Endoscopic score (in the bowel segment with the most severe lesions)^a^	≤1	0
	>1	1
Perianal lesions	No	0
	Yes	1
Complications: stenosis, any type of fistula, extraintestinal manifestations or fever >38 °C	No	0
	Yes (≥1)	1

Abbreviations: CRP, C-reactive protein; PreMiCC, prediction score for a mild course of Crohn’s disease.

^a^Endoscopic score: 0, no lesions; 1, some aphthous lesions or small ulcerations (< 5); 2, aphthous lesions and ulcerations (> 5), some areas with normal mucosa; 3, aphthous lesions and ulcerations and diffuse mucosal inflammation; and 4, diffuse large ulcerations and/or stenosis.

After patients’ initial therapy was adapted to their disease severity and individual circumstances, their escalation status was assessed at week 8 after the baseline visit (Figure 1). If any of the following conditions occurred, a patient was considered to have escalated treatment at week 8: (1) received therapy other than mesalazine; (2) were not steroid-free for 2 weeks; (3) received immunosuppressants or biologics; or (4) underwent CD-related surgery. The inception cohort of all 201 patients with CD, whether or not their therapy was escalated, was enrolled in the follow-up analysis. The follow-up visits occurred every 3 months for the first year and then every 6 months until 3 years and then annually for up to 5 years. Outcome failures were defined as (1) a necessary therapeutic escalation with steroids or oral budesonide, (2) the initiation of immunosuppressive or biologic therapy, or (3) IBD-related hospitalization or surgery.

The primary objective of the present prospective, real-world, observational study was to test and validate our PreMiCC score. This score is based on simple and readily available clinical parameters and aims to predict a mild course vs a more severe course of CD.^11^ A secondary study objective was to determine the overall proportion of CD patients experiencing a mild course in this inception cohort.

### Documentation and Statistical Analysis

Findings of the clinical examinations and laboratory parameters (CRP, white blood cell count, hemoglobin, ferritin, and stool examinations) were documented in the baseline and follow-up questionnaires. The findings of the endoscopic examinations, such as ileocolonoscopy, including biopsies, were also documented. Nevertheless, because this was a noninterventional study, these examinations were only performed based on patient needs and the decision of each treating physician.

Continuous normally distributed variables were reported as means and 95% confidence intervals, and skewed variables as medians and interquartile ranges. Categorical variables were presented as percentages. For categorical data, the chi-square test and Fisher’s exact test were used. If the assumptions for parametric testing were not met, the Wilcoxon rank sum test was used to compare numeric outcomes between the 2 groups. A *P* value below .05 was considered statistically significant. Qualitative measures are described using absolute and percentage frequencies. The log-rank test was used to examine whether the divergence of the Kaplan-Meier curves was statistically significant. IBM SPSS Statistics 27 was used for statistical analysis.

### Ethical Statement

All patients were required to provide written informed consent. The study was approved by the ethics committee of the Medical Association of Westphalia-Lippe and the University of Münster (AZ: 2014-064-f-S). The study was registered at ClinicalTrials.gov (NCT02193048). All data collected in the practices were anonymized and stored in a secure electronic database. Documentation was performed using a secure data server hosted by an independent organization (IOMTech GmbH, Berlin; DomainFactory GmbH, computing center in Cologne, Germany).

## Results

The prospective, noninterventional PROGNOS study included 201 CD patients with a diagnosis made in IBD-specialized practices in Germany less than 6 weeks before being enrolled in the study. The 201 CD patients comprising the study cohort were assigned using the previously described score (PreMiCC)^11^ at baseline to 2 subgroups: those with a score ≤2 (ie, who were likely to have a mild course) and those with a score >2 (ie, who were likely to have an unfavorable course of disease). At baseline, 88 were assigned a score ≤2 and 113 a score >2 (Table 1). The baseline characteristics of the patients are shown in Table 2, including the score parameters, according to their assignment to 1 of the 2 groups with a PreMiCC score ≤2 or >2. There were differences between the 2 groups in the baseline characteristics depending on the score assignment. Patients in the ≤2 score group were more likely to be older, have lower CRP and endoscopic scores, and have fewer complications, stenoses, and steroid therapies. However, no significant differences were observed between the 2 groups with regard to diarrhea or abdominal pain at baseline.

**Table 2.** Baseline characteristics, including the score parameters, depending on assignment to the 2 groups with a PreMiCC score ≤2 or >2

Characteristics at first diagnosis	Score >2		Score ≤2		Total	
	Findings	n	Findings	n	Findings	n
**Score**	4	113	1	88	3	201
**Age**, y	29	113	46^a^	88	33	201
≤40 y	93 (82.3)	—	34 (38.6)	—	127 (63.2)	—
**Female**	63 (55.8)	113	51 (58)	88	114 (56.7)	201
**CRP**, mg/dL	14.4	113	1.4^a^	88	5.6	201
<2 mg/dL	3 (2.7)	—	53 (60.2)	—	56 (27.9)	—
2-4 mg/dL	13 (11.5)	—	24 (27.3)	—	37 (18.4)	—
>4 mg/dL	97 (85.8)	—	11 (12.5)	—	108 (53.7)	—
**Fecal calprotectin**, mg/kg	355	69	74	54	208	123
**Bowel resection at first diagnosis**	2 (1.8)	113	0 (0)	88	2 (1)	201
**Endoscopic score** >1	93 (82.3)	113	33 (37.5)^a^	88	126 (62,7)	201
No lesions	1 (0.9)	—	3 (3.4)	—	4 (2.0)	—
Some aphthous lesions/no ulcerations (< 5)	18 (15.9)	—	52 (59.1)	—	70 (34.8)	—
Aphthous lesions and ulcerations >5, normal mucosa	39 (34.5)	—	23 (26.1)	—	62 (30.8)	—
Aphthous lesions/ulcerations and diffuse mucosal inflammation	31 (28.3)	—	9 (10.2)	—	40 (20.4)	—
Diffuse large ulcerations and/or stenosis	23 (20.4)	—	1 (1.1)	—	24 (11.9)	—
**Upper intestinal tract affected**: no	71 (62.8)	113	48 (54.5)	88	119 (59.2)	201
**Upper intestinal tract affected**: yes	12 (10.6)	—	2 (2.3)	—	14 (7)	—
**Upper intestinal tract affected**: not investigated	30 (26.5)	—	38 (43.2)	—	68 (33.8)	—
**Perianal lesions**	10 (8.8)	113	1 (1.1)	88	1 (1.1)	201
**Complications**	27 (23.9)	113	1 (1.1)^a^	88	28 (13.9)	201
**Stenosis**	20 (17.7)	113	1 (1.1)^a^	88	21 (10.4)	201
**Extraintestinal manifestation**	7 (6.2)	113	0 (0)	88	7 (3.5)	201
**Fistulas**	6 (5.3)	113	0 (0)	88	6 (3)	201
**Fever >38 °C**	2 (1.8)	113	0 (0)	88	2 (1)	201
**Prior treatment for CD**	47 (41.6)	113	17 (19.3)	88	64 (31.8)	201
**Mesalazine administration**	20 (17.7)	113	11 (12.5)	88	31 (15.4)	201
**Steroids**	36 (31.9)	113	10 (11.4)^a^	88	46 (22.9)	201
**Immunosuppressants/biologics**	0 (0)	113	0 (0)	88	0 (0)	201

Values are median or n (%), unless otherwise indicated.

Abbreviations: CD, Crohn’s disease; CRP, C-reactive protein; PreMiCC, prediction score for a mild course of Crohn’s disease.

^a^Significant difference for univariate testing between a PreMiCC score >2 and ≤2.

**Figure 2.**
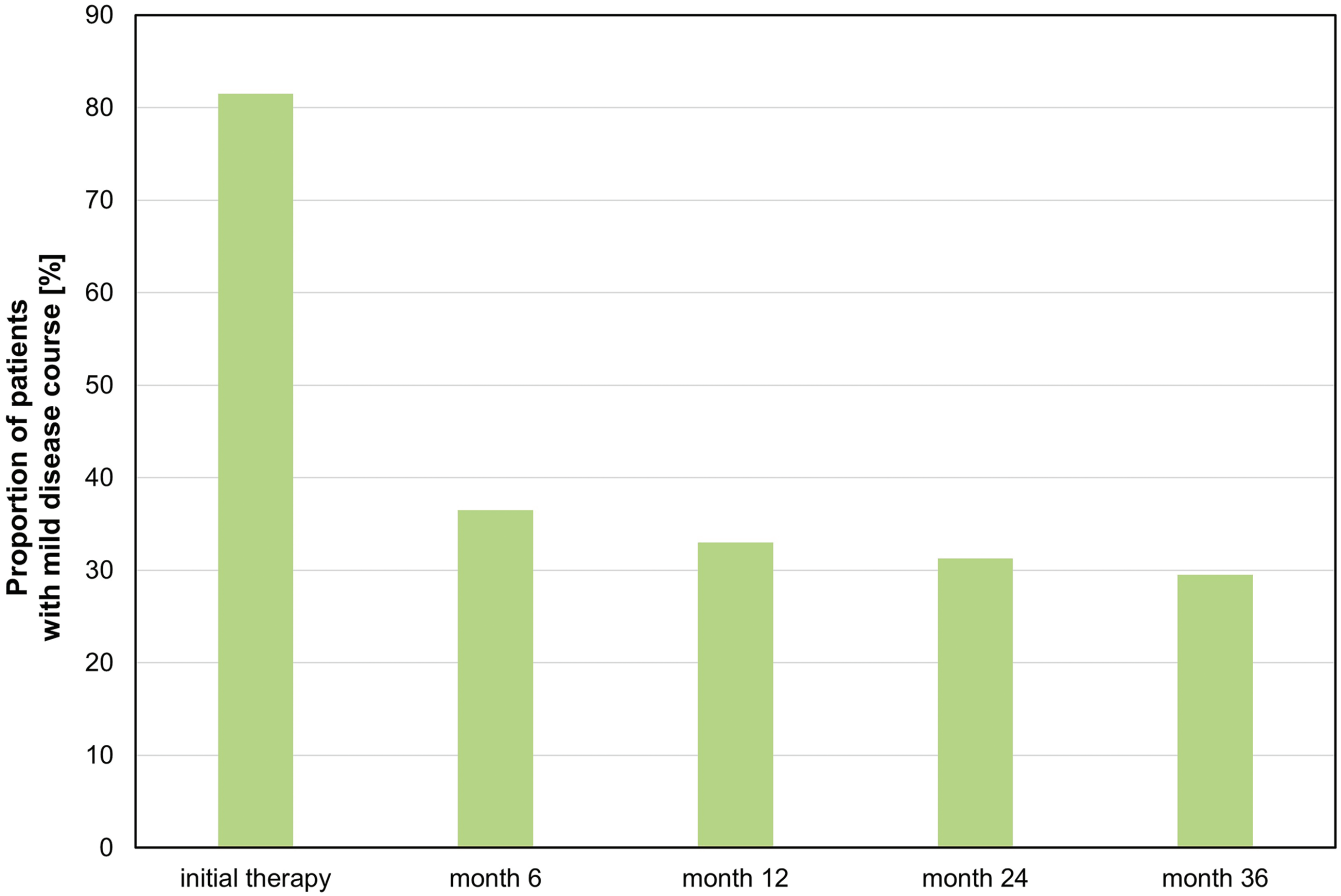
Proportion of patients with a mild course of disease without therapy escalation in the entire inception cohort of patients newly diagnosed with Crohn’s disease after initial therapy (8 weeks) and after 6, 12, 24, and 36 months (Kaplan-Meier rates).

At week 8, after their inclusion in the study and starting an initial, individually tailored therapy, a total of 19.5% of the 201 CD patients (19.6% in the group with a score >2 and 16.9% in the group with a score ≤2) had escalated their therapy according to our predefined criteria of therapy escalation (Figure 2).

At 3 years, 29.5% of patients had a mild course of CD with no need for treatment escalation. Most of the necessary escalations occurred at the beginning (ie, within the first 6 months after starting their initial therapy) (Figure 2).

**Figure 3.**
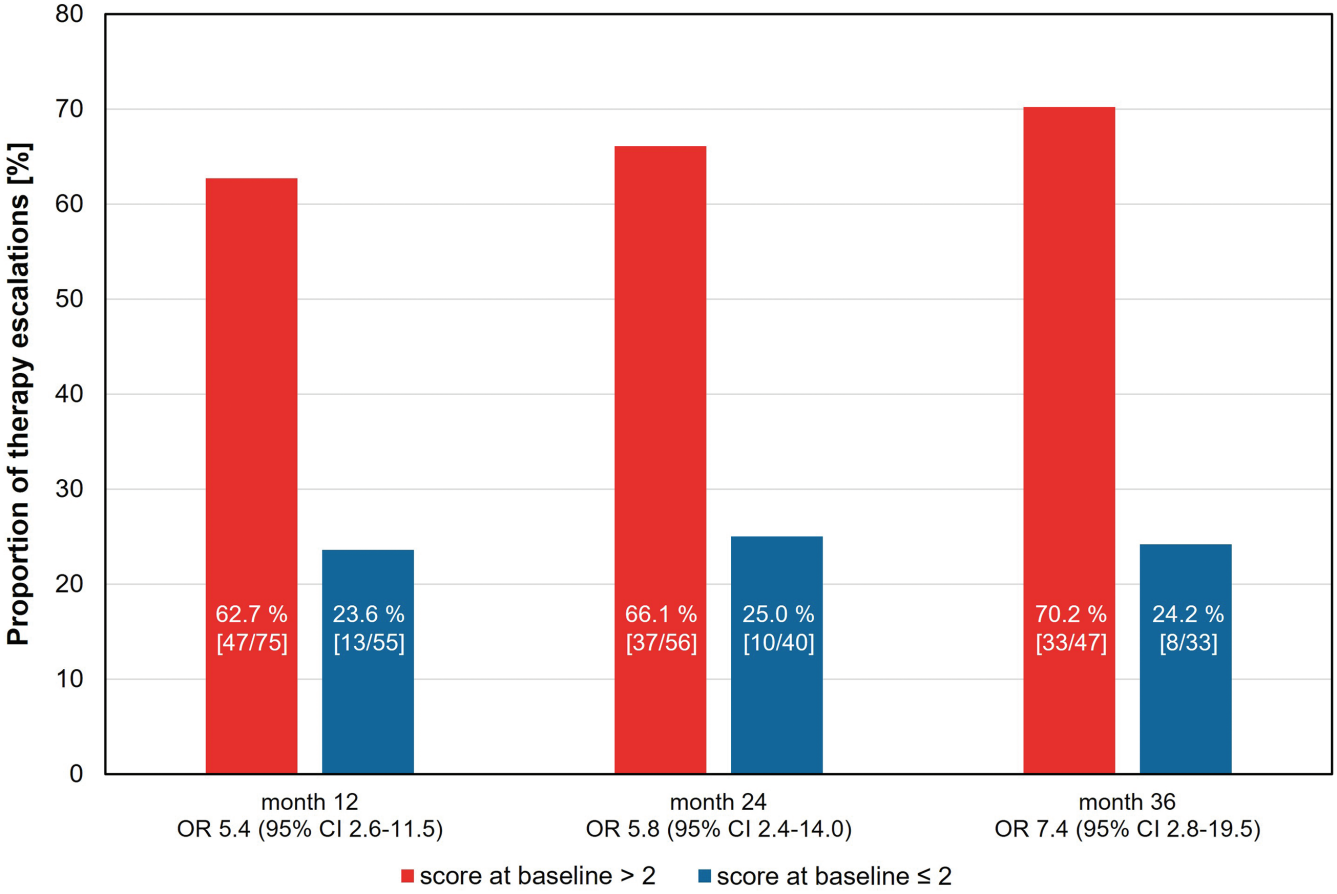
Proportion of therapy escalations per score class after 12, 24, and 36 months with odds ratios (ORs) and 95% confidence intervals (CIs).

In total, the 2 groups were compared over a mean observation period of 38.4 months. Among those with a score ≤2, therapy escalation at 36 months was necessary for only 24.2%, whereas in the group with a score >2, Significantly more therapy escalations were necessary for 70.2% of patients in the as-observed analyses (Figure 3).

These differences between the 2 groups were also found with regard to the biological therapies initiated over the course of the study. After 3 years, 46.8% of patients in the group with a PreMiCC score >2 and only 9.1% of patients with a PreMiCC score ≤2 had initiated treatment with biologics. A similar picture was observed for surgery (12.0% vs 0.0%) and hospitalization (12.0% vs 0.0%) after 3 years.

**Figure 4.**
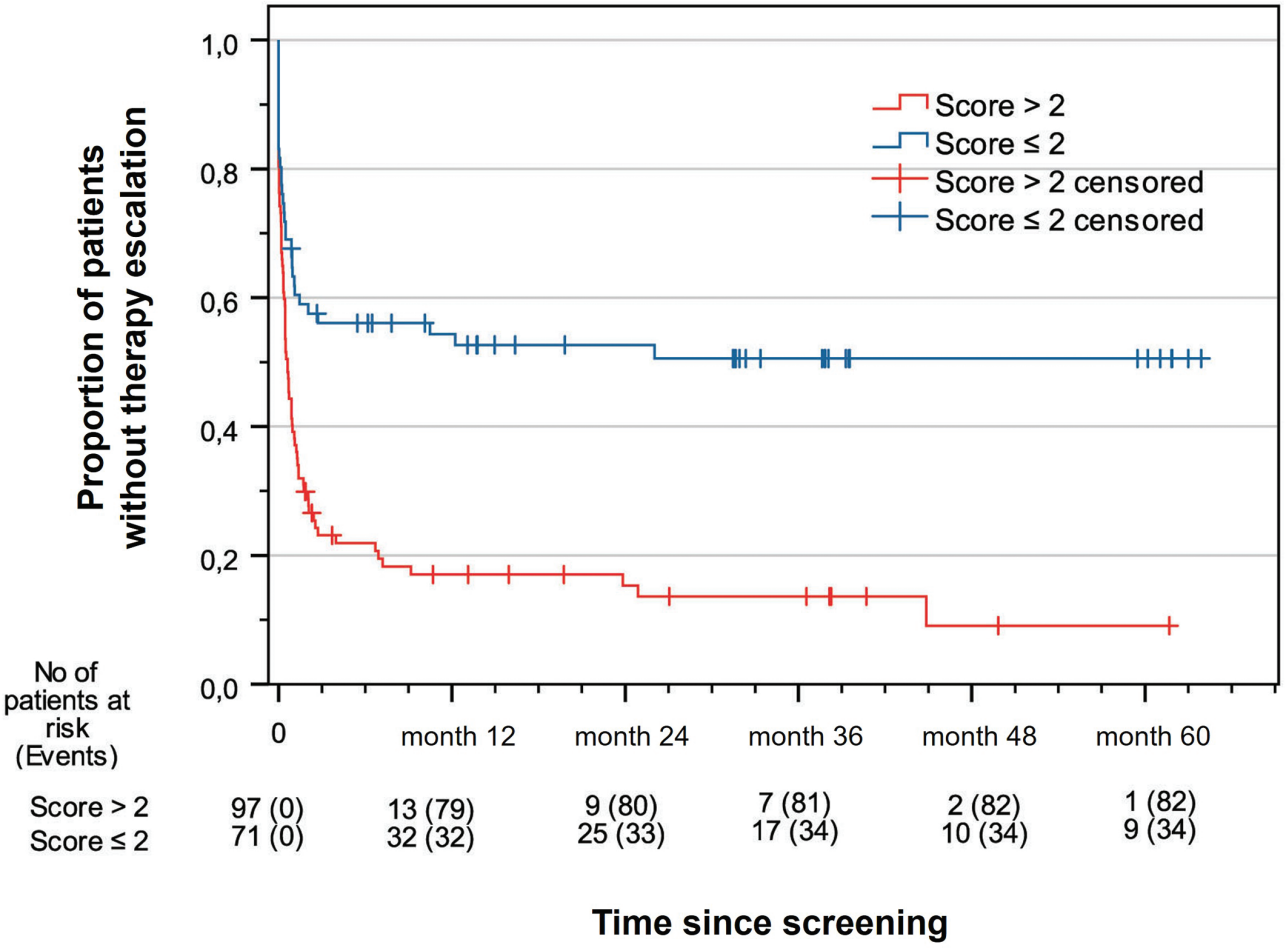
Kaplan-Meier plot for time to therapy escalation in Crohn’s disease patients with PreMiCC (prediction score for a mild course of Crohn’s disease) >2 or ≤2.

**Figure 5.**
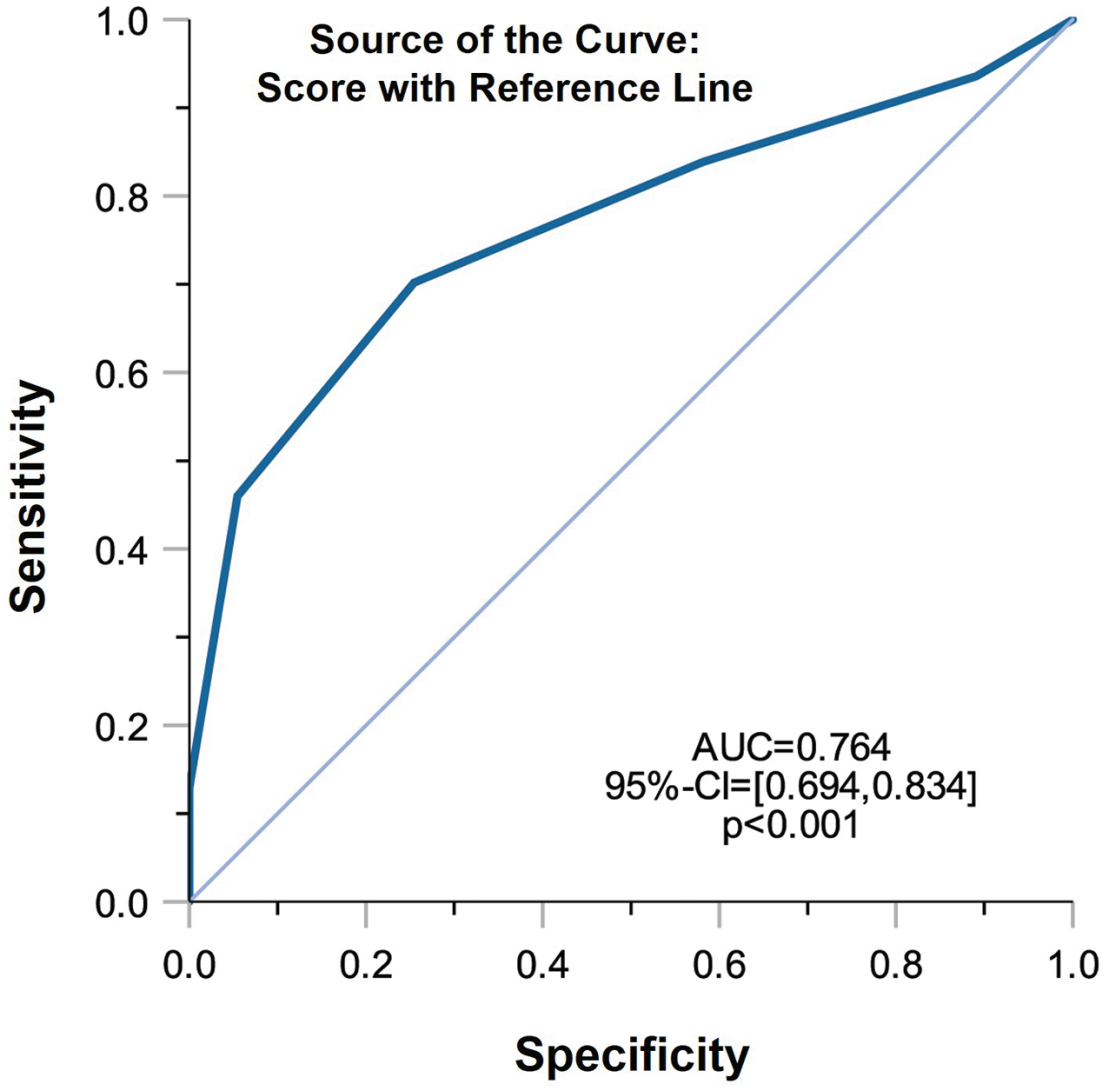
Receiver-operating characteristic curves of the PreMiCC (prediction score for a mild course of Crohn’s disease) (score ≤2 vs >2). AUC, area under the curve.

The Kaplan-Meier curve (Figure 4) shows that a significantly larger proportion of patients who did not escalate therapy were in the group with a PreMiCC score ≤2. There was a pronounced and statistically significant divergence of the curves starting at 3 months and extending to 48 months (*P* < .001). Thus, at 48 months, the positive predictive value of the PreMiCC for correctly predicting a mild course was 80.0%, with a sensitivity of 90.9% and a specificity of 78.3%. The high sensitivity and specificity can also be seen in the receiver-operating characteristic curve (area under the curve, 0.764; *P* < .001) (Figure 5).

## Discussion

In our inception cohort of patients in Germany diagnosed with CD in gastroenterology practices specialized in IBD, approximately 30% of patients were found to have a mild course of disease over a follow-up period of up to 5 years. This real-world finding of a substantial proportion of CD patients with a mild course is in concordance with other studies, which have also found an uncomplicated course (ie, in terms of nonprogressive disease) in 20% to 30% of patients.^8-10,12^ Another population-based inception cohort study of unselected CD patients in Europe^23^ reported that under mesalazine monotherapy in the first year, 16% of patients had a mild course. In our study, among the patients with a PreMiCC score ≤2 (indicating a mild course of disease) who showed a need for treatment escalation, therapy escalation was more frequent during the first 12 weeks but was followed by a relatively stable course. In both groups, disease complications or the first increase in disease activity requiring therapy escalation predominantly occurred within the first year after diagnosis. In other words, patients’ treatment could be adapted on demand, avoiding chronic intestinal damage, during clinical visits following the diagnosis. Despite the unclear efficacy of mesalazine in CD, most of these patients continued to have a markedly mild course of disease over the entire follow-up period of up to 5 years and could be treated with mesalazine alone without therapy escalation. Another population-based study, from the Netherlands, also found that a substantial proportion of patients followed over 10 years had a favorable course under therapy with mesalazine alone.^24^ While the results of a recent study from Israel suggest that there was no difference between mesalazine therapy and no therapy during the maintenance phase, it should be noted the study was based on registry data that were not differentiated in terms of mild or severe CD.^25^ In a Danish cohort, a similar frequency of mild progression under mesalazine in CD patients was shown.^26^

In contrast to severe CD with its typical complications, such as strictures, fistulas, or the need for surgery or long-term steroid treatment, there are no consistently established definitions of a mild, nonprogressive course of CD. Various publications have described the main criteria for a mild (nonprogressive) course as the absence of the complications described previously, a lack of a need for treatment escalation beyond conventional therapy, and maintenance of a mild course without long-term steroid treatment.^13^

Data on factors predictive of a mild course of CD are scarce. Some studies mention higher age and a less complicated course of disease at diagnosis as possible criteria.^23^ As a diagnostic alternative, a digital calculator for assessing the likelihood of a more indolent disease course in newly diagnosed CD patients is of interest.^12^ However, it does not seem to be easy to implement in real-world clinical settings, partly because it lacks clear cut-off values. In contrast, our PreMiCC^11^ relies on relatively simple and readily available information about the clinical picture of a patient early in the diagnostic process. According to the findings of the present study, the score can distinguish between patients with a rather mild course and those with a severe course from an unselected group of CD patients at a very early stage of diagnosis. Indeed, here we show the ability of the PreMiCC to distinguish between the 2 groups in the very first months after diagnosis (Figure 4), similar to the findings of Yanai et al.^12^ In our study, as well as in that of Yanai et al, the vast majority of therapy escalations occurred in the first 3 to 6 months after diagnosis.

Nevertheless, given that some cases are acute and severe, top-down therapy using immunosuppressants and biologics in the earlier phase of CD has been suggested as a possible approach to treating CD in severe cases.^27,28^ This may have the advantage of fewer hospitalizations and surgeries among this population, but a clinically relevant benefit for individual patients of early treatment with immunosuppressants and biologics has not yet been clearly demonstrated in well-designed interventional studies. In addition, the number of treat-to-target goals for the management of CD patients has increased substantially, meaning that patients are now monitored very closely and therapy is escalated, where necessary, in a timely manner.^29^ Even with this trend toward closer monitoring and therapy adjustment, however, the necessary type and intensity of initial therapy for patients with mild symptomatology have not been defined to date. In this regard, our proposed score shows good discrimination between severe and milder courses of disease along with clinical parameters that are readily available early in the diagnostic process. According to the disease course shown in Figure 4 (Kaplan-Meier plot for time to therapy escalation), we suggest very close monitoring during the first 3 months after the beginning of the treatment with clinical examination, laboratory examination, and, if necessary, additional abdominal ultrasound and calprotectin, while subsequent intervals of 3 to 4 months seem to be appropriate. In this way, patients with a milder course can be effectively managed with mesalazine monotherapy or, if necessary, with rapid treatment escalations. Such close monitoring would also help with decisions about escalating therapy if the conventional therapy used initially should ultimately prove to be inadequate.

There has been much discussion about possible approaches to treating CD patients who have a mild course of disease. In contrast to the published findings of some studies,^26,30,31^ most major clinical guidelines^3,22^ do not recommend the use of aminosalicylates in CD, taking into account the predominantly negative results of this drug in clinical trials in CD patients as a whole. However, it should be noted that such trials have not been carried out in a placebo-controlled manner, especially not in patients who have a mild course of disease and are treated with mesalazine.

In the present study, we successfully validated the proposed PreMiCC score for assessing a mild or severe course of CD based on our previous work.^11^ Being able to use the results of the PreMiCC score at diagnosis to distinguish between a rather mild course of CD and a severe course means that physicians can identify patients in whom, for example, mesalazine alone and close monitoring may be sufficient. This can help avoid unnecessary therapy escalation with immunosuppressants or biologics, sparing patients the risk of side effects under these treatments.

The PROGNOS study has several strengths and weaknesses that must be considered when interpreting its results. One strength is that the patients in this cohort were prospectively enrolled and had a confirmed diagnosis, with the initial diagnosis not having been made earlier than 6 weeks before study inclusion. Moreover, all patients were followed prospectively for up to 5 years (mean 38.4 months). Another strength lies in the fact that the baseline characteristics of our cohort mirror those of the population of CD patients who receive an initial diagnosis in IBD-specialized gastroenterology practices across Germany. However, this could also represent a limitation, as some extremely severe cases might not have been included in our study cohort because they would have required immediate hospital treatment, thus bypassing the IBD-specialized practices participating in our study. Such cases are, however, probably very rare given that the primary diagnosis of CD in Germany almost always takes place in IBD-specialized practices.^11,32^ Another limitation of the study is that the number of patients in the cohort was not very high, but this was unavoidable from a logistical perspective given that the patients had to be enrolled at a very early stage of disease; moreover, previous studies of CD patients with a mild disease course have had even smaller cohorts. A further limitation is that the PreMiCC score was only validated in a German cohort in this study; further studies in other countries with different healthcare systems could also be helpful. Another limitation concerns the endoscopic component of the score, which was not formally validated in clinical practice. We adopted this component based on the Rutgeerts score for CD with surgery because it has proven itself in that context, it is pragmatic to use, and there is still no practical and easy-to-use score for assessing endoscopic findings in CD. Moreover, among patients with a mild course in our cohort, it was not possible to differentiate between those who received mesalazine alone or possibly no CD therapy at all due to the small group sizes, as it is a widespread practice in Europe to treat patients with a milder course with mesalazine alone. Last, another limitation is that the physicians at the participating practices did not have to follow a standardized study protocol for therapy or other interventions in this noninterventional study, but rather tailored their approach based on disease severity and individual circumstances.

## Conclusions

In this prospective study of the course of incident CD, approximately 30% of patients were found to have a mild disease course without a need for immunosuppressive/biologic therapy over up to 3 years of follow-up. Furthermore, we were able to validate the ability of our proposed PreMiCC score to predict which patients would go on to have a mild course of disease during the study period. Further prospective studies with larger cohorts of patients with a mild course of disease are warranted to confirm these findings.

## Data Availability

The data analyzed in this study cannot be shared publicly due to the privacy of participants. The data will be shared upon reasonable request made to the corresponding author.
